# Supplementary material for: N-Glycomics of Human Erythrocytes
Source: Int J Mol Sci. 2021 Jul 28;22(15):8063. doi: 10.3390/ijms22158063 (PMC8347577; doi:10.3390/ijms22158063)
Supplement: Supplementary file 1 [file ijms-22-08063-s001.zip › N-Glycomics of human erythrocytes_Supplementary Figures 1-12.pdf]

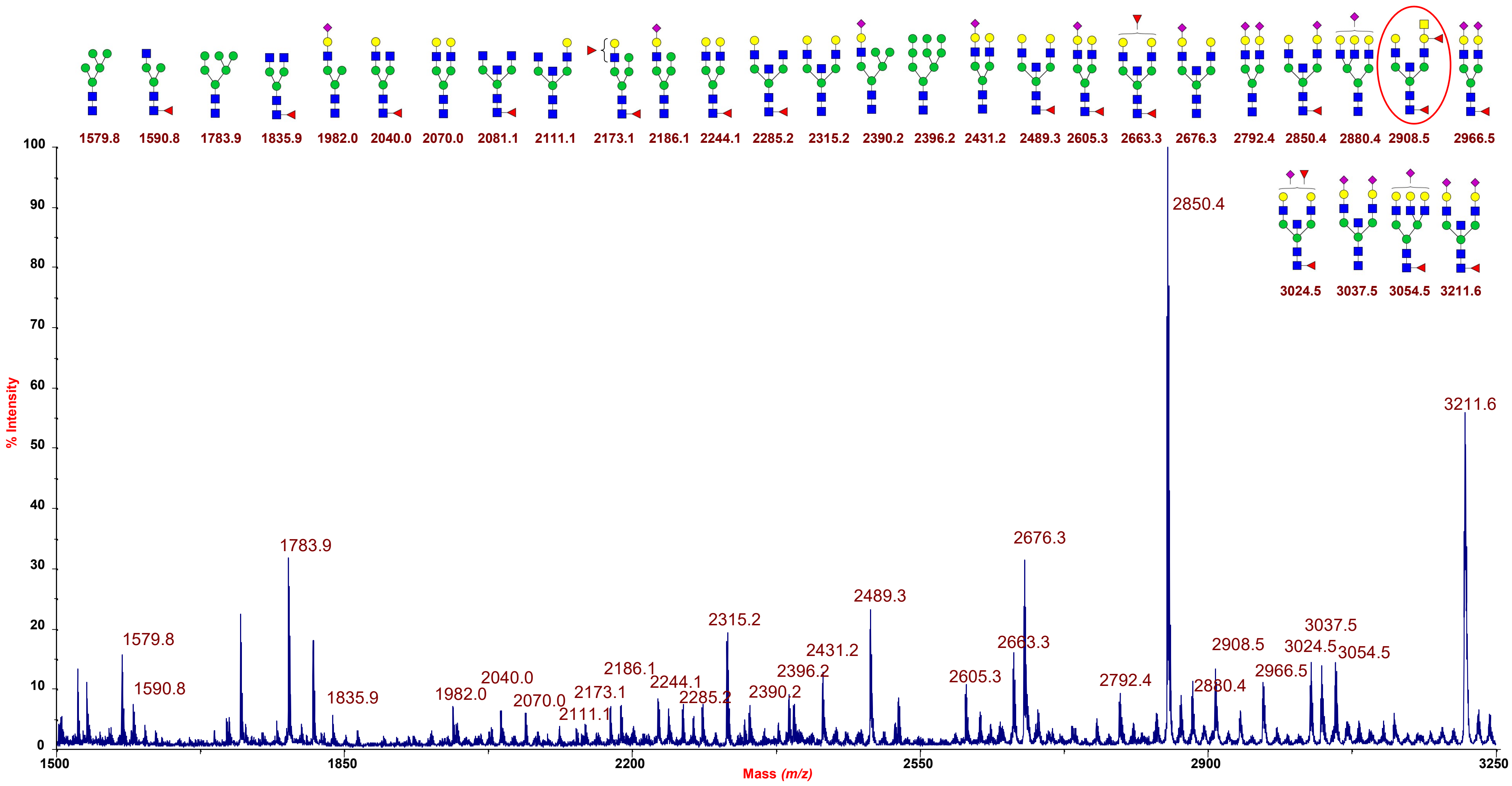

Supplementary Figure 1. MALDI-TOF MS profile (low mass-range: *m/z* 1500-3250) of permethylated human erythrocyte N-glycans from blood group A
The present spectrum shows significant assignments, with those structures comprising the blood group A epitope highlighted with red circles.
GlcNAc: blue square; Man, green circle; Gal, yellow circle; NeuAc, purple lozenge; Fuc, red triangle.

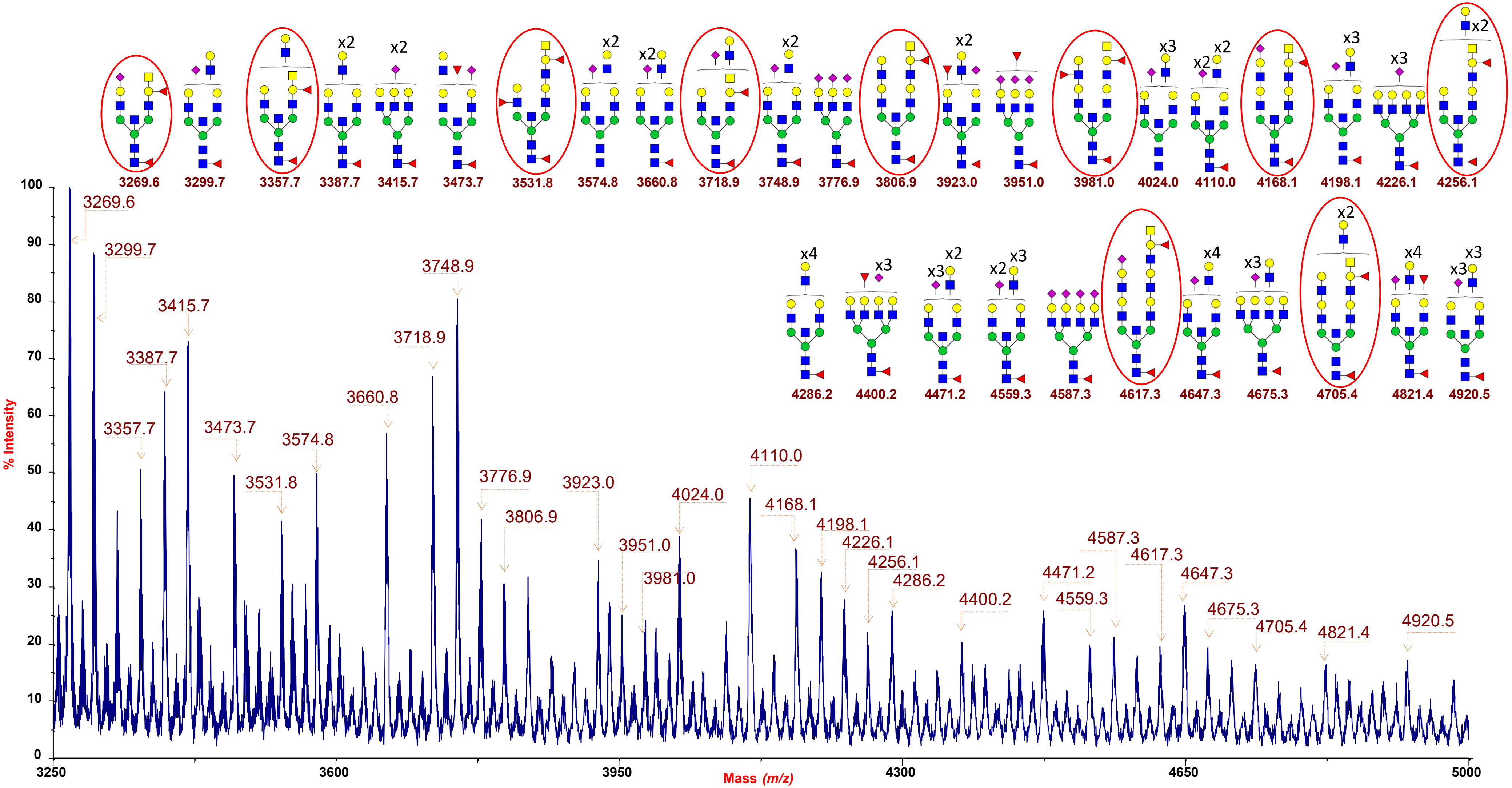

Supplementary Figure 2. MALDI-TOF MS profile (middle mass-range: *m/z* 3250-5000) of permethylated human erythrocyte N-glycans from blood group A

The present spectrum shows significant assignments, with those structures comprising the blood group A epitope highlighted with red circles.

GlcNAc: blue square; Man, green circle; Gal, yellow circle; NeuAc, purple lozenge; Fuc, red triangle.

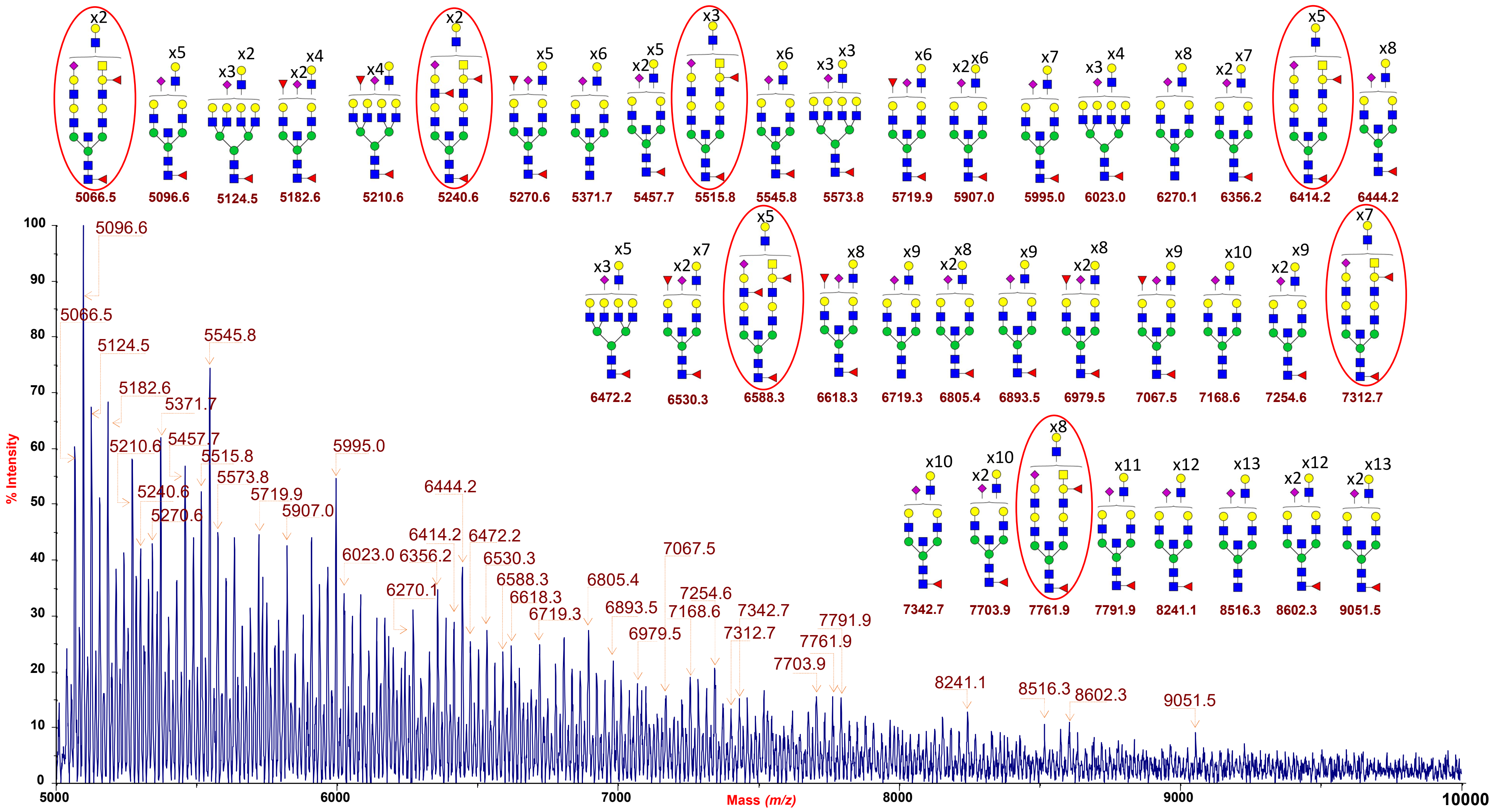

Supplementary Figure 3. MALDI-TOF MS profile (high mass-range: *m/z* 5000-10000) of permethylated human erythrocyte N-glycans from blood group A

The present spectrum shows significant assignments, with those structures comprising the blood group A epitope highlighted with red circles.

GlcNAc: blue square; Man, green circle; Gal, yellow circle; NeuAc, purple lozenge; Fuc, red triangle.

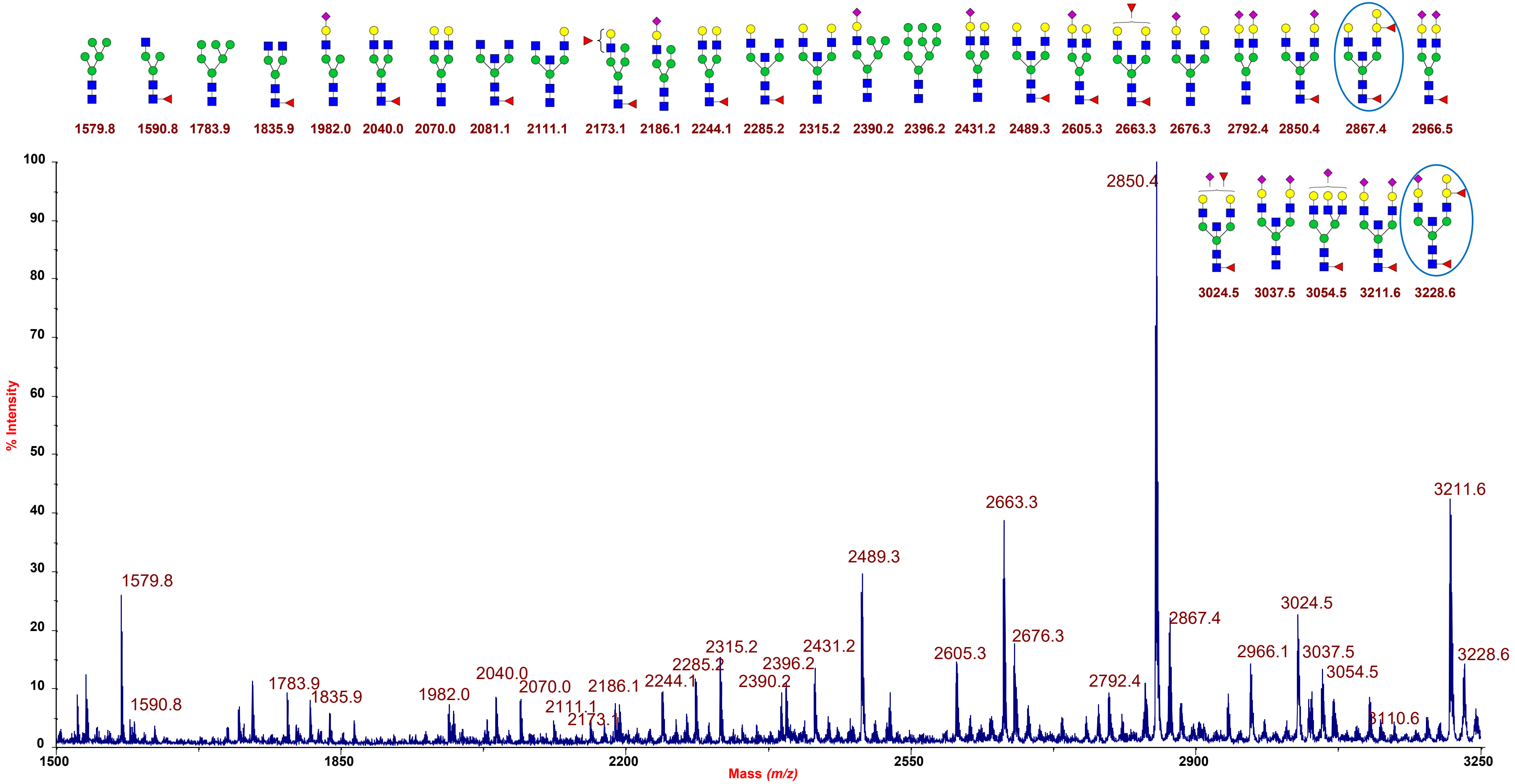

Supplementary Figure 4. MALDI-TOF MS profile (low mass-range: *m/z* 1500-3250) of permethylated human erythrocyte N-glycans from blood group B
The present spectrum shows significant assignments, with those structures comprising the blood group B epitope highlighted with blue circles.
GlcNAc: blue square; Man, green circle; Gal, yellow circle; NeuAc, purple lozenge; Fuc, red triangle.

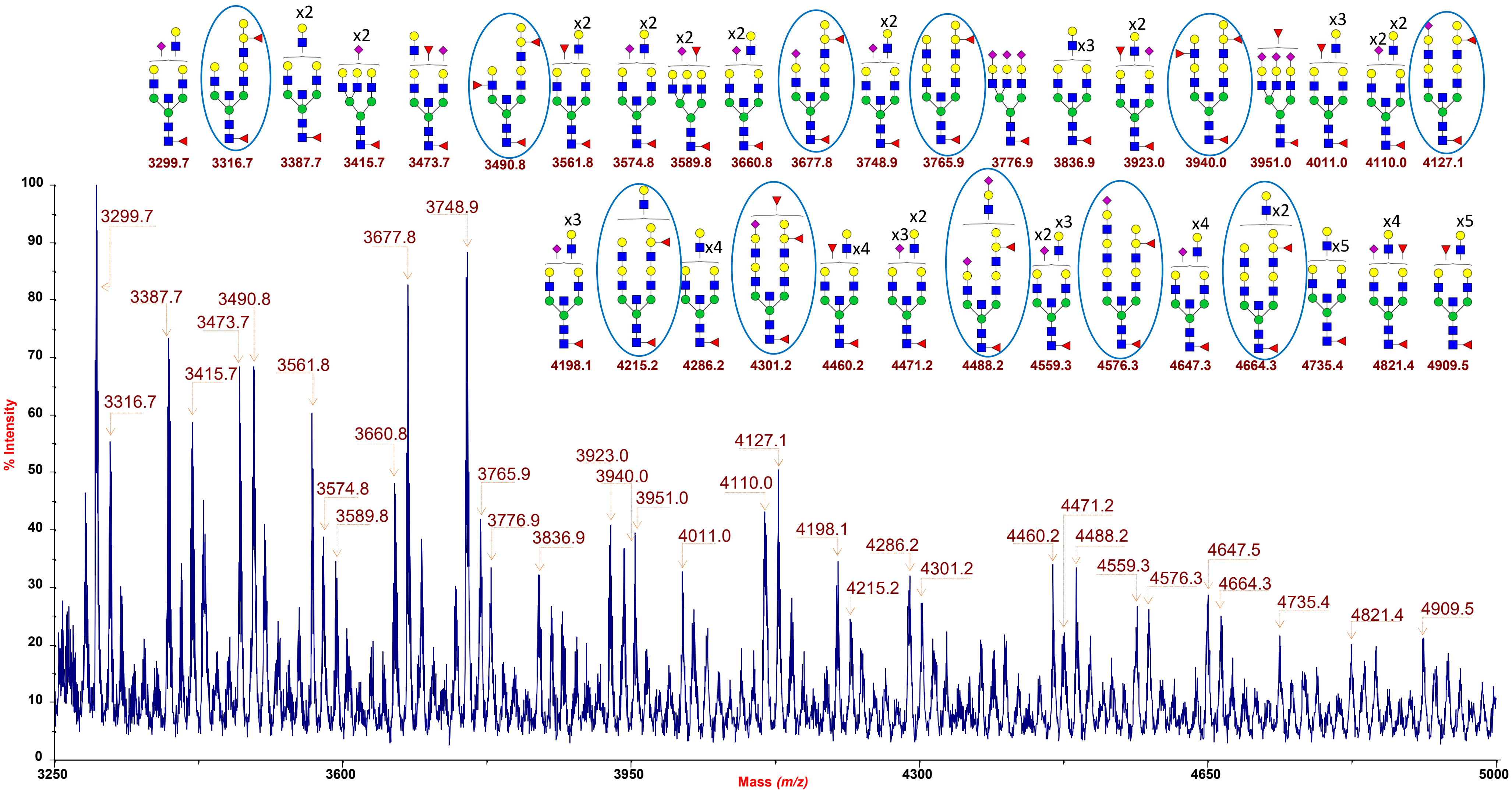

Supplementary Figure 5. MALDI-TOF MS profile (middle mass-range: *m/z* 3250-5000) of permethylated human erythrocyte N-glycans from blood group B

The present spectrum shows significant assignments, with those structures comprising the blood group B epitope highlighted with blue circles.

GlcNAc: blue square; Man, green circle; Gal, yellow circle; NeuAc, purple lozenge; Fuc, red triangle.

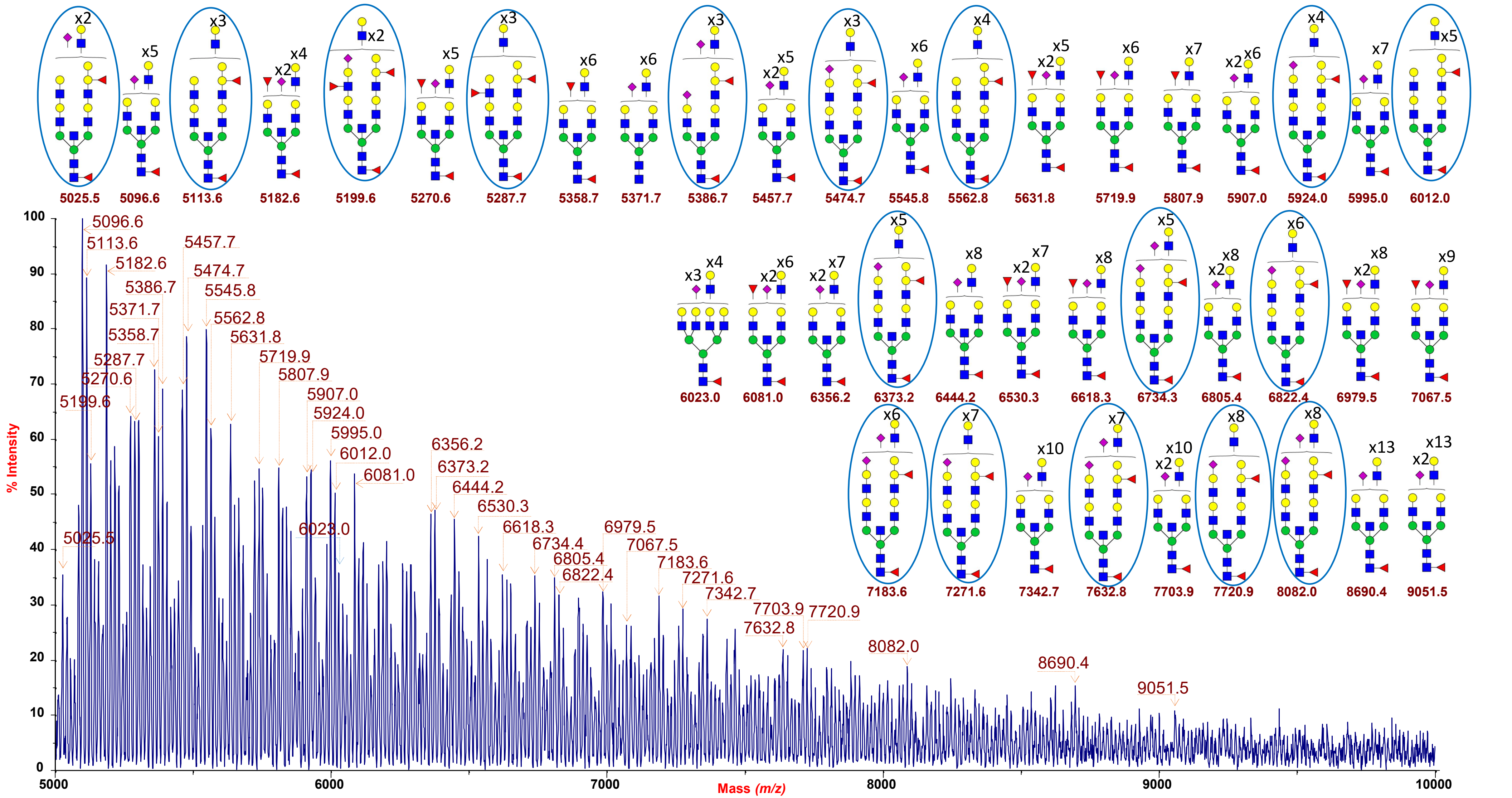

Supplementary Figure 6. MALDI-TOF MS profile (high mass-range: *m/z* 5000-10000) of permethylated human erythrocyte N-glycans from blood group B
The present spectrum shows significant assignments, with those structures comprising the blood group B epitope highlighted with blue circles.
GlcNAc: blue square; Man, green circle; Gal, yellow circle; NeuAc, purple lozenge; Fuc, red triangle.

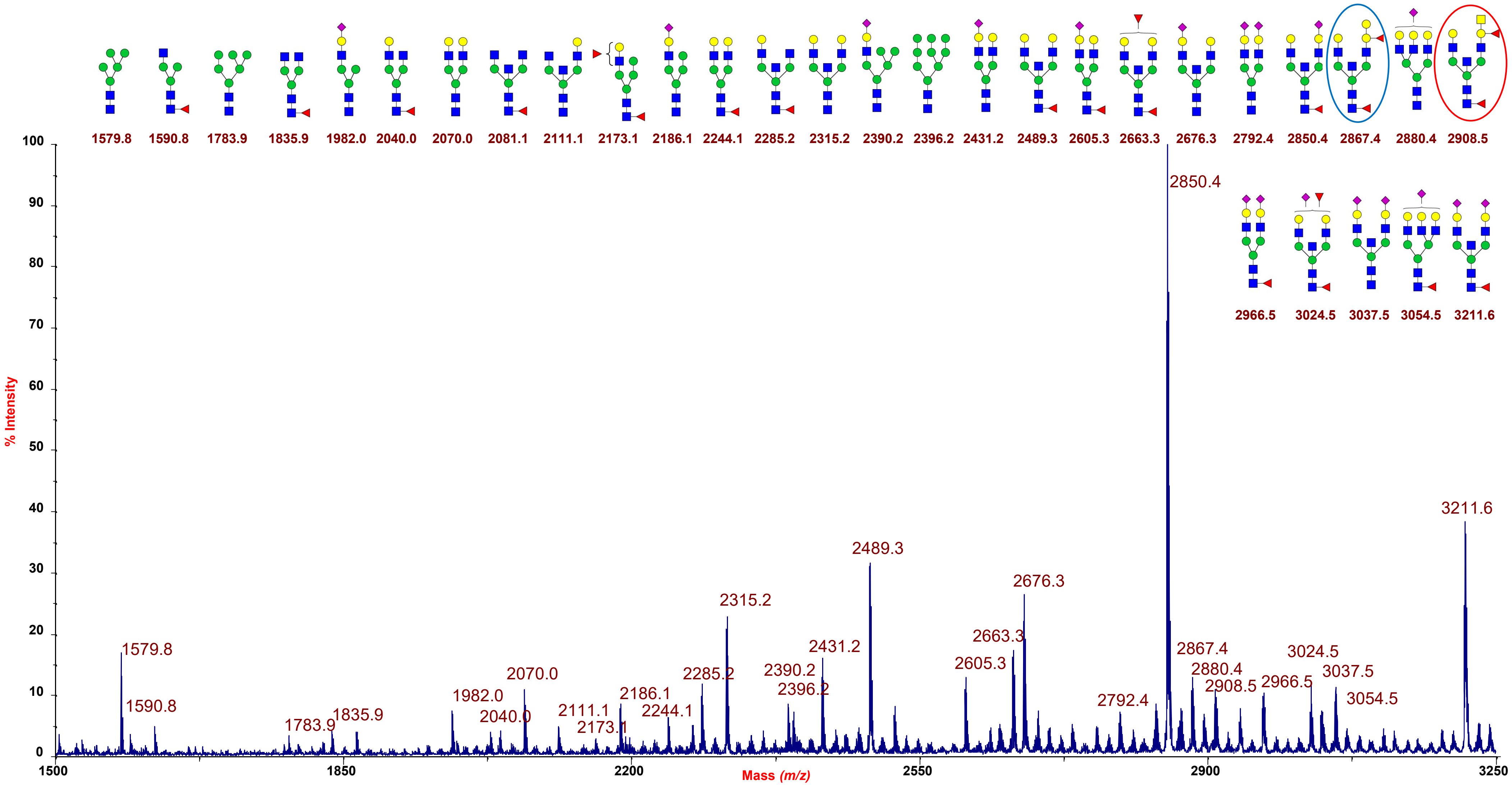

Supplementary Figure 7. MALDI-TOF MS profile (low mass-range: *m/z* 1500-3250) of permethylated human erythrocyte N-glycans from blood group AB
The present spectrum shows significant assignments, with those structures comprising blood group A or B epitopes highlighted with red or blue circles, respectively. GlcNAc: blue square; Man, green circle; Gal, yellow circle; NeuAc, purple lozenge; Fuc, red triangle.

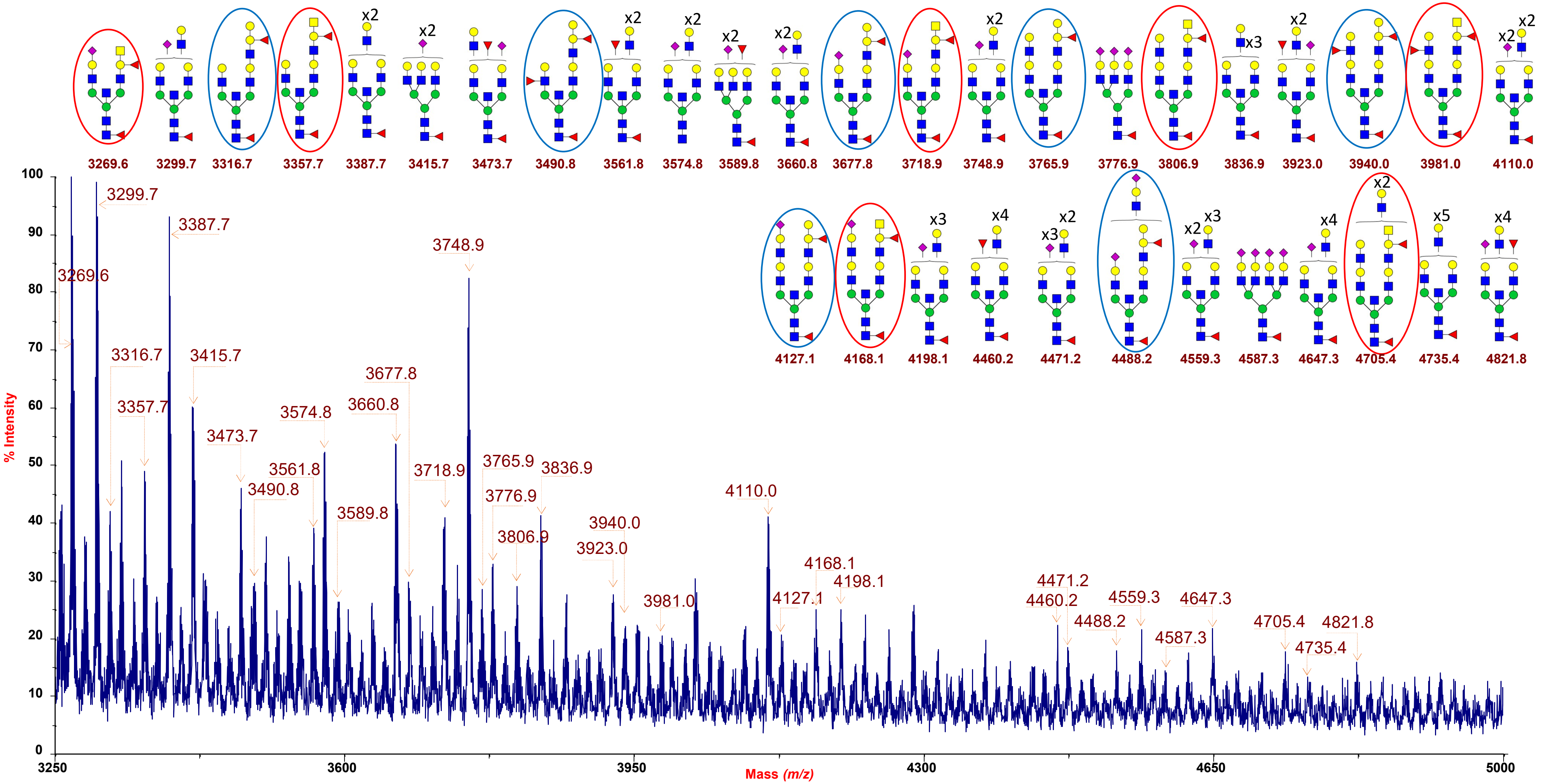

Supplementary Figure 8. MALDI-TOF MS profile (middle mass-range: *m/z* 3250-5000) of permethylated human erythrocyte N-glycans from blood group AB
The present spectrum shows significant assignments, with those structures comprising blood group A or B epitopes highlighted with red or blue circles, respectively. GlcNAc: blue square; Man, green circle; Gal, yellow circle; NeuAc, purple lozenge; Fuc, red triangle.

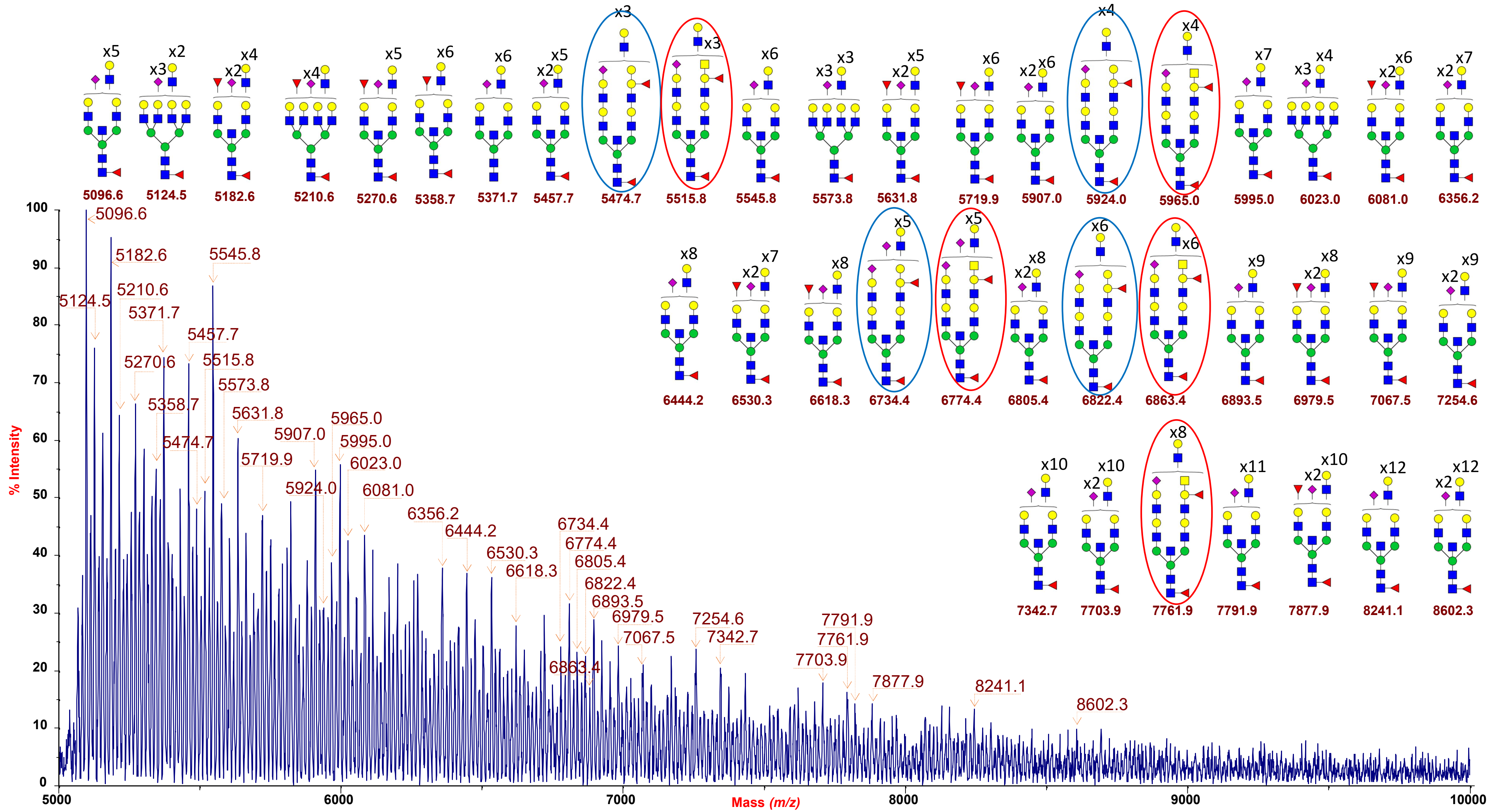

Supplementary Figure 9. MALDI-TOF MS profile (high mass-range: *m/z* 5000-10000) of permethylated human erythrocyte N-glycans from blood group AB

The present spectrum shows significant assignments, with those structures comprising blood group A or B epitopes highlighted with red or blue circles, respectively. GlcNAc: blue square; Man, green circle; Gal, yellow circle; NeuAc, purple lozenge; Fuc, red triangle.

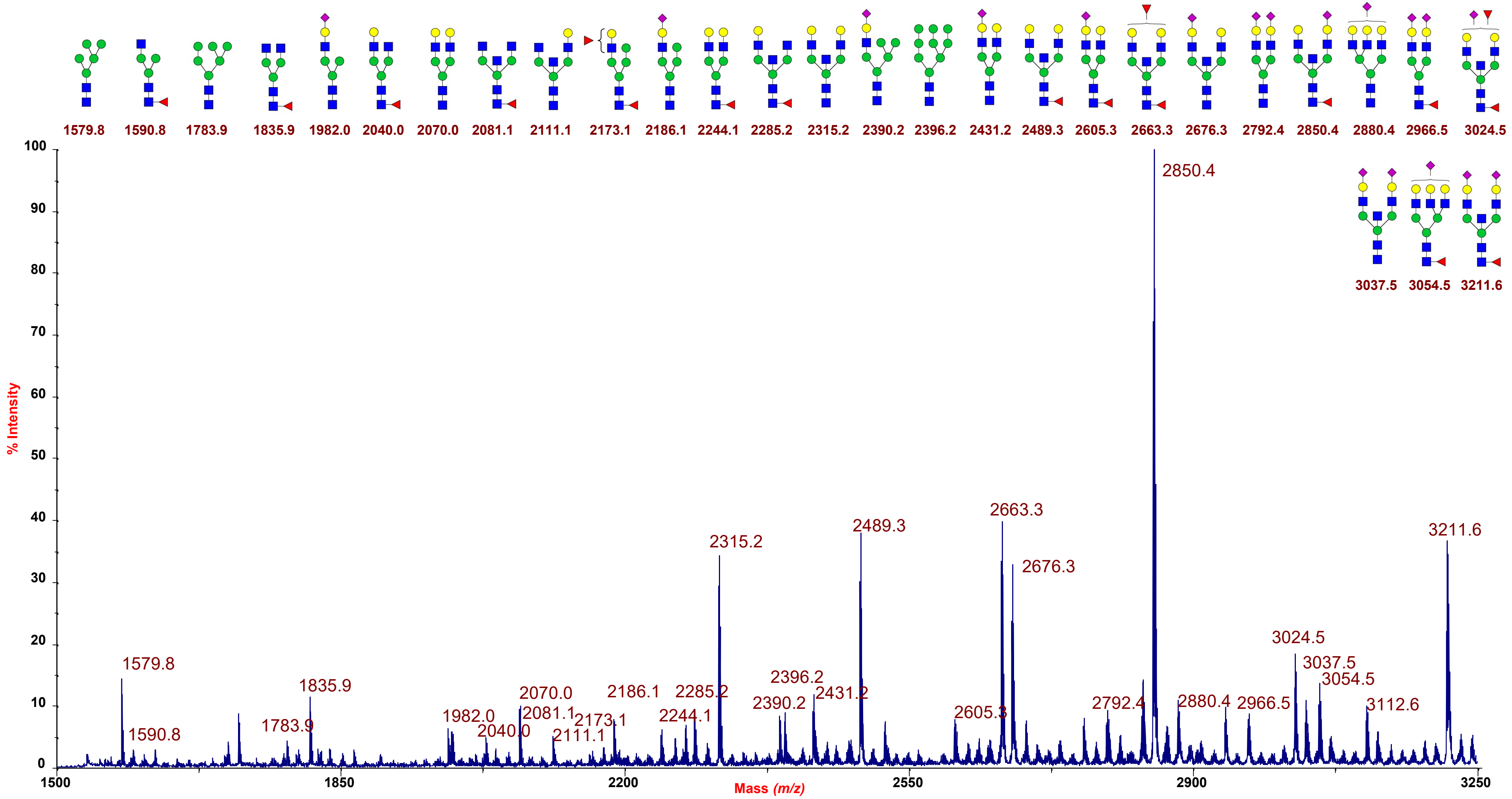

Supplementary Figure 10. MALDI-TOF MS profile (low mass-range: *m/z* 1500-3250) of permethylated human erythrocyte N-glycans from blood group O

The present spectrum shows significant assignments characterized by the absence of distinctive H antigen-bearing structures.

GlcNAc: blue square; Man, green circle; Gal, yellow circle; NeuAc, purple lozenge; Fuc, red triangle.

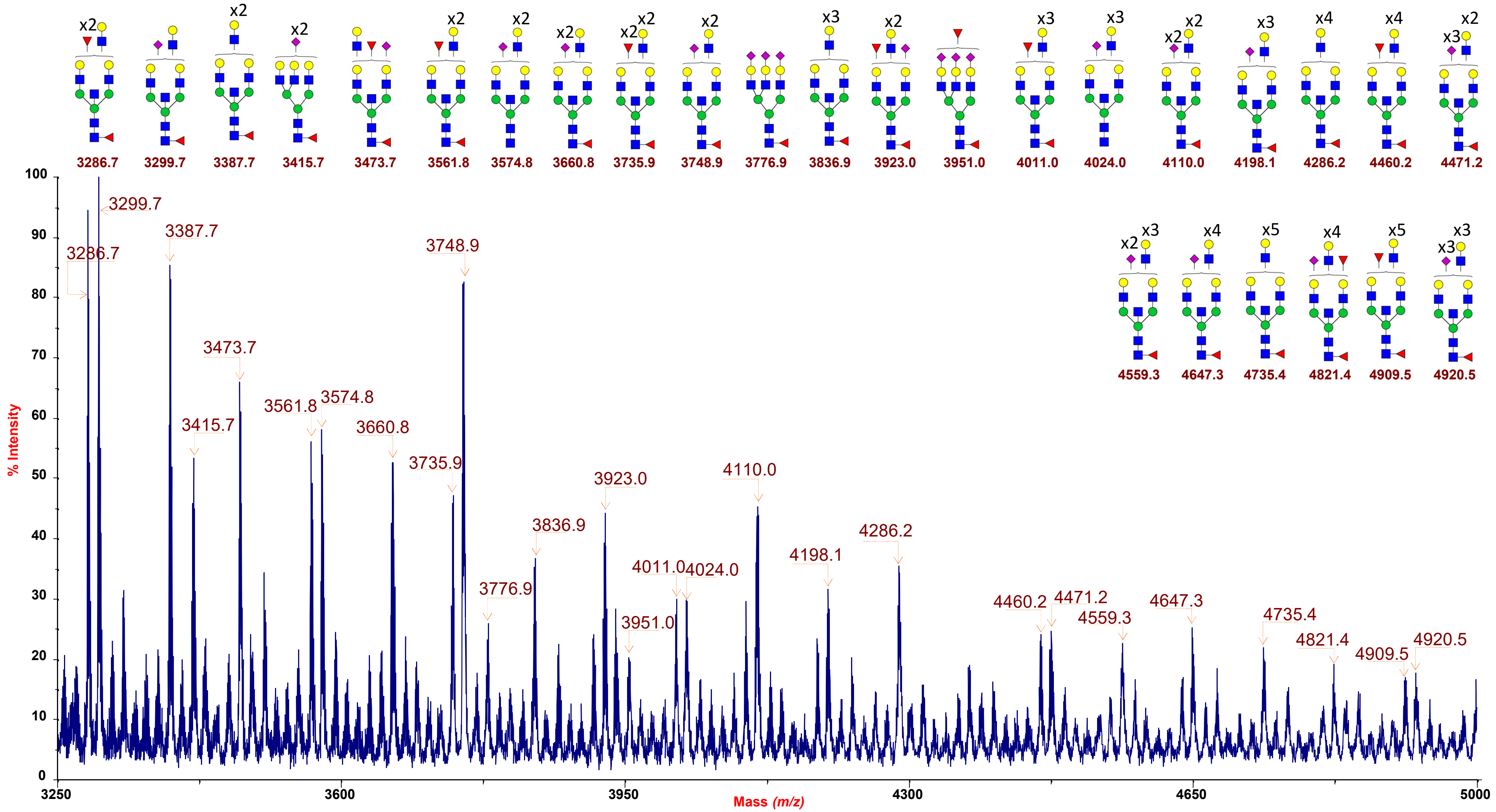

Supplementary Figure 11. MALDI-TOF MS profile (middle mass-range: *m/z* 3250-5000) of permethylated human erythrocyte N-glycans from blood group O
The present spectrum shows significant assignments characterized by the absence of distinctive H antigen-bearing structures.
GlcNAc: blue square; Man, green circle; Gal, yellow circle; NeuAc, purple lozenge; Fuc, red triangle.

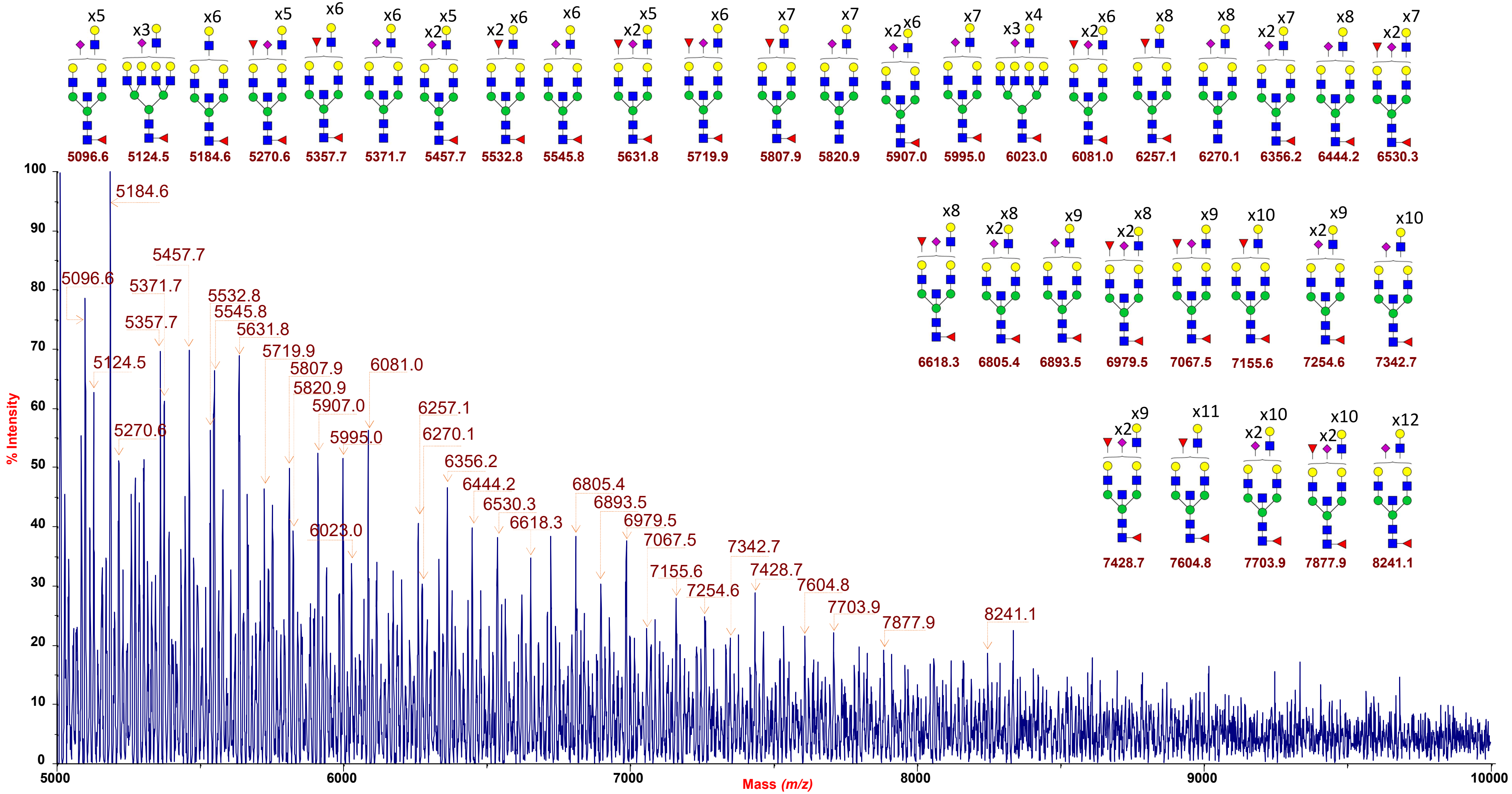

Supplementary Figure 12. MALDI-TOF MS profile (high mass-range: *m/z* 5000-10000) of permethylated human erythrocyte N-glycans from blood group O

The present spectrum shows significant assignments characterized by the absence of distinctive H antigen-bearing structures.

GlcNAc: blue square; Man, green circle; Gal, yellow circle; NeuAc, purple lozenge; Fuc, red triangle.
